# Peanut skin bioactive extract on pectin and gelatin candies: is it a potential dietary antioxidant delivery system for health improvement?

**DOI:** 10.1002/jsfa.70230

**Published:** 2025-10-03

**Authors:** Luiza Gabriella Soares Dantas Pinheiro, Ana Carla de Matos, Gabriela de Matuoka e Chiocchetti, Priscilla Efraim, Gabriela Alves Macedo, Juliana Alves Macedo

**Affiliations:** ^1^ Department of Food and Nutrition Faculty of Food Engineering, State University of Campinas – UNICAMP Campinas Brazil; ^2^ Department of Food Technology Faculty of Food Engineering, State University of Campinas – UNICAMP Campinas Brazil

**Keywords:** peanut skin extract, gummy candies, phenolic compounds, antioxidant capacity

## Abstract

**BACKGROUND:**

Peanuts (*Arachis hypogaea* L.) are widely consumed worldwide, with Brazil being a major producer. Their skins, usually discarded as processing waste, comprising about 3% of the total peanut weight, generated an estimated 1.50 Mt of waste in 2021. These skins are rich in phenolic compounds, including phenolic acids and flavonoids, with antioxidant capacities ranging from 90 to 125 mg gallic acid equivalents per gram dry weight. In this study, a phenolic‐rich extract was prepared from peanut skins and used to enrich pectin‐ and gelatin‐based candies at 0.1 and 0.2 g kg^−1^. The candies underwent static *in vitro* digestion following the INFOGEST protocol, covering the oral, gastric and intestinal phases.

**RESULTS:**

Bioaccessibility of total phenolic compounds was 29.88–32.46% for 0.1 and 0.2 g kg^−1^ pectin‐based candies and 25.94–30.16% for 0.1 and 0.2 g kg^−1^ gelatin‐based candies. Regarding antioxidant activity after digestion, pectin gummy candies showed an average reduction of approximately 38%, while gelatin gummy candies exhibited a decrease of nearly 41%, measured by oxygen radical absorbance capacity. Nonetheless, the candies preserved a considerable portion of their original antioxidant capacity, even after digestion.

**CONCLUSION:**

The results highlight the promising potential of using peanut skin phenolic extracts to produce antioxidant‐enriched gummy candies. This approach offers a healthier alternative for consumers and promotes sustainability by valorizing abundant local agricultural by‐products. Future studies could explore strategies to improve bioaccessibility, such as micro‐ or nano‐encapsulation, and process optimization further to enhance the stability and bioavailability of these compounds. © 2025 The Author(s). *Journal of the Science of Food and Agriculture* published by John Wiley & Sons Ltd on behalf of Society of Chemical Industry.

## INTRODUCTION

Peanut (*Arachis hypogaea* L.) is an oilseed legume widely adaptable to different climates and soils, allowing cultivation in various tropical regions. It has a significant economic impact on the global agricultural sector. It ranks as the fourth‐largest oilseed crop globally, after soybean, cotton and rapeseed.^1^ In Latin America, Brazil is the second‐largest producer, with cultivation especially strong in São Paulo, where production reached 726.4 thousand tons in 2022 and continues to grow.^2^

At the same time that peanut production is high, a significant amount of waste is also generated. Currently, peanut skins, which represent approximately 3% of the total peanut weight, are mostly discarded as processing waste. Standard disposal methods include landfilling, incineration and use as animal feed, all of which can contribute to environmental pollution and resource depletion. Therefore, developing sustainable alternatives for the valorization of peanut skins is crucial to minimize environmental impact and support circular economy practices in the agroindustry.^3^, ^4^

Nevertheless, these residues present a promising opportunity when value is attributed to them, as peanut skins are a rich and viable source of bioactive compounds, particularly phenolic compounds such as flavonoids and phenolic acids.^5^ This potential paves the way for the commercial exploitation of these valuable constituents in peanut skins.

Thus, using peanut skin extract as a functional food ingredient offers a health‐focused alternative to conventional inputs. It adds value to local agriculture by reusing an abundant, low‐cost by‐product, reducing raw material costs, promoting sustainability, closing the production cycle, and fostering technological and economic development in agribusiness.

Gummy candies are confections made with sugar syrups and gelling agents like gelatin, starch or pectin.^6^ Their popularity has grown due to the demand for products with natural and functional ingredients. Thanks to their shelf stability and simple processing, gummies are promising vehicles for bioactive delivery. Pectin, derived from orange juice by‐products in Brazil, is a sustainable, plant‐based gelling agent. Gelatin, widely accessible and often enriched with collagen, appeals to health‐conscious consumers.^7^

Among the food matrices suitable for incorporating bioactive phenolic extracts, gummy candies stand out in line with current confectionery trends. They were selected as the delivery system for peanut skin extract due to their broad consumer appeal and favorable physicochemical characteristics for phenolic preservation, such as low water activity and dense texture.^8^ Furthermore, their complex flavor profile can effectively mask the pronounced astringency typically associated with phenolic compounds.^9^ The gummy candy market, particularly pectin‐based products, has grown steadily in Brazil and internationally, driven by increasing demand for natural, sustainable and functional formulations.^7^, ^8^, ^10^, ^11^

In light of the above, the study aimed to develop gummy candies as carrier systems for extracts rich in phenolic compounds from peanut processing industry waste, assessing their antioxidant potential after interaction with the matrix and the bioaccessibility of bioactive compounds in *in vitro* models.

## METHODOLOGY

### Sample acquisition and extract preparation

Peanut skins (Valencia variety), provided by Ampendupã (Tupã, SP, Brazil), were ground, sieved (10‐mesh), and stored at −80 °C. Phenolic compounds were extracted following a previously optimized method developed by our research group,^12^ based on an experimental design approach. Briefly, 50 g of sample was mixed with 125 mL ethanol and 125 mL water, stirred (200 rpm, 25 °C, 2 h), filtered under vacuum using Whatman No. 1 filter paper, and concentrated via rotary evaporation (40 °C, 2 h). The residue was resuspended in 100 mL water, frozen, lyophilized and stored at −80 °C.

### Confection of pectin and gelatin candies

Pectin and gelatin‐based gummy candies were prepared according to previously described methods.^13^ Table 1 shows the formulations. The amounts of water, glucose syrup, pectin, citric acid and sodium citrate were kept constant across all samples. The sucrose content was reduced proportionally to the amount of freeze‐dried peanut skin extract added by a simple weight substitution, meaning that for every gram of extract incorporated, 1 g sucrose was removed, thereby maintaining the total solids content of the formulation. Three pectin‐based candies were produced: control without extract (P0); enriched with 0.1 g kg^−1^ extract (P1); and enriched with 0.2 g kg^−1^ extract (P2). Similarly, three gelatin‐based candies were prepared: control without extract (G0); enriched with 0.1 g kg^−1^ extract (G1); and enriched with 0.2 g kg^−1^ extract (G2).

**Table 1:** Ingredients for control and peanut skin extract‐enriched pectin and gelatin candies

Ingredients (%)	Pectin candies			Gelatin candies		
	P0	P1	P2	G0	G1	G2
Water	28	28	28	28	28	28
Sucrose^a^	35	34	33	35	34	33
Glucose syrup^b^	35	35	35	35	35	35
Pectin^c^ or gelatin^d^	15	15	15	15	15	15
Extract	0	1	2	0	1	2
Citric acid^e^	0.35	0.35	0.35	0.35	0.35	0.35
Sodium citrate^f^	0.15	0.15	0.15	0.15	0.15	0.15

^a^ Refined sugar (Union, Sao Paulo, Brazil).

^b^ Glucose syrup 40 DE (Excel 1040, Ingredion, Sao Paulo, Brazil).

^c^ Pectin (GENU CPKelco, Minneapolis, USA).

^d^ SiMoGel (Rousselot, Angouleme, France).

^e^ Citric acid anhydrous (Cargill, Sao Paulo, Brazil).

^f^ Sodium citrate (Cargill, Sao Paulo, Brazil). P0, pectin candy without extract; P1, pectin candy with 0.1 g kg^−1^ extract; P2, pectin candy with 0.2 g kg^−1^ extract; G0, gelatin candy without extract; G1, gelatin candy with 0.1 g kg^−1^ extract; G2, gelatin candy with 0.2 g kg^−1^ extract.

**Figure 1:**
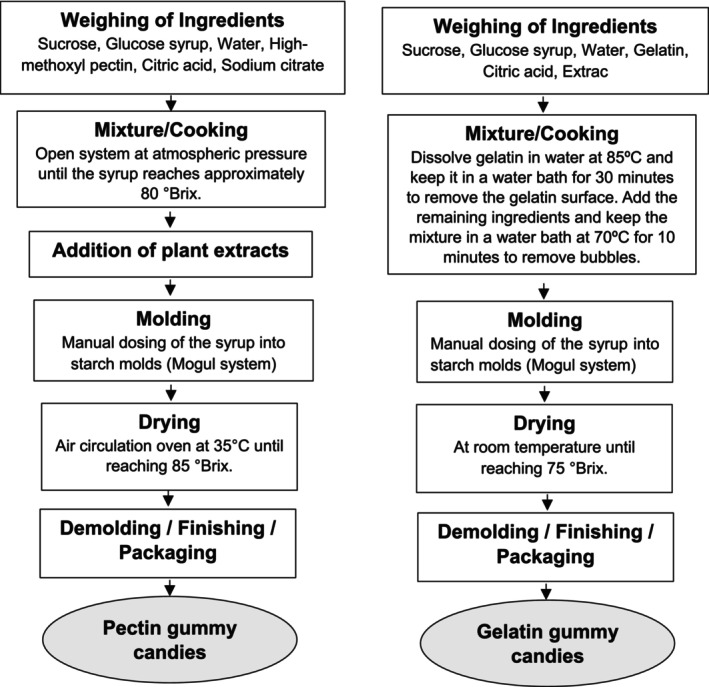
Flowchart of the production process of pectin and gelatin gummy candies.

The pectin gummy candies were developed following the steps presented in Fig. 1: (1) dissolving sucrose and glucose syrup in cold water: (2) dispersing pectin in the sugar solution; (3) cooking the syrup under atmospheric pressure until it reaches 80 °Brix; (4) adding the peanut skin extract powder, citric acid and sodium citrate; (5) depositing the syrup into starch molds and drying in an oven with air circulation (model TE‐394/2, Tecnal, Piracicaba, Brazil) at 35 °C; (6) demolding, finishing and packaging the candies.

The gelatin candies were produced following these steps: (1) dissolving the gelatin in water at 85 °C, maintaining it in a water bath at 70 °C for 30 min, with removal of a film formed on the surface of the gelatin; (2) adding the remaining ingredients to the gelatin; (3) cooking in a water bath at 70 °C for 10 min to remove bubbles; (4) depositing the syrup into starch molds and drying at room temperature until reaching 85 °Brix; (6) demolding, finishing and packaging the candies.

The processing flowcharts of pectin and gelatin gummy candies, developed in the present study, are shown in Fig. 1.

### Physical–chemical analysis of the candies

The physical–chemical properties analyzed included water activity, moisture, °Brix, texture (hardness and viscosity) and color.

Water activity was determined in triplicate using a dew point analyzer (Aqualab 4TEV, Decagon Devices, Pullman, WA, USA) at 25 °C.^14^ Moisture content was measured by vacuum oven drying at 105 °C following AOAC 920.151.^14^ °Brix was analyzed in triplicate using a digital refractometer (model r^2^i300, Reichart Technologies, Depew, NY, USA) after molding.^14^ Texture was evaluated with a TA.XT2i texture analyzer (Stable Micro Systems, Godalming, UK), compressing samples twice with an acrylic disc at 1.0 mm s^−1^ and 0.01 N trigger force.^15^, ^16^ The determination was conducted in ten replicates to measure the hardness and viscosity of the samples. Color (L*, a*, b*) was measured with a digital colorimeter (UltraScan PRO, HunterLab, Reston, VA, USA) in nine replicates.^17^, ^18^

### Antioxidant delivery potential evaluation

#### Total phenolics determination before and after *in vitro* gastrointestinal digestion

The total phenolic content (TPC) was determined in triplicate using the Folin–Ciocâlteu method,^19^ with modifications. For undigested candies, 5 g was heated at 37 °C to yield 5 mL of solution (S1), followed by 1:50 and 1:100 dilutions. For digested samples, 1 mL aliquots were collected at the end of each digestive phase (oral, gastric, intestinal) and diluted as needed.

In both cases, 50 μL of the sample was mixed with 800 μL distilled water, 50 μL Folin–Ciocâlteu reagent and, after 3 min, 100 μL Na_2_CO_3_ solution. The reaction proceeded for 2 h at room temperature in the dark. Absorbance was measured at 725 nm (model DU 640, Beckman Coulter, Brea, CA, USA). Results were calculated from a gallic acid standard curve constructed from seven dilutions ranging from 15 to 300 μg mL^−1^ and expressed as milligrams of gallic acid equivalents (GAE) per gram of candy.

#### Oxygen radical absorbance capacity assay before and after digestion

The oxygen radical absorbance capacity (ORAC) assay was performed as previously described,^20^ using undigested candies and samples from the intestinal phase post‐digestion. Undigested samples (S1) were diluted 1:50 and 1:100 in 75 mmol L^−1^ phosphate buffer (pH 7.4), and digested samples were diluted 1:5. In 96‐well plates, 120 μL of 70 nmol L^−1^ fluorescein and 20 μL of sample were mixed, followed by 60 μL of 12 mmol L^−1^ AAPH (2,2′‐azobis(2‐amidinopropane) dihydrochloride). Trolox (30–1500 μmol mL^−1^) was used for calibration.

Readings were taken at 37 °C (excitation: 485 nm; emission: 520 nm) on a FLUOstar OPTIMA fluorimeter (BMG Labtech, Germany). A blank (standard candy without extract) was subtracted. Results were expressed as millimoles of Trolox equivalents (TE) per gram of candy.

### Simulated *in vitro* digestion of the prepared candies

A standardized static *in vitro* digestion was performed based on the INFOGEST protocol,^21^, ^22^ which is widely recognized for simulating human gastrointestinal digestion. For the oral phase, 5 g candy was incubated at 37 °C for 2 min with simulated salivary fluid (pH 7.0) and α‐amylase (75 U mL−1; final volume: 10 mL). The gastric phase followed, adjusting pH to 3.0 and adding simulated gastric fluid with pepsin (2000 U mL^−1^), incubated at 37 °C for 2 h (final volume: 18 mL). Then, pH was adjusted to 7.0, and the intestinal phase was carried out with pancreatin (100 U mL^−1^) and bile salts (10 mmol L^−1^; final volume: 26 mL). Enzyme concentrations and conditions strictly followed the INFOGEST guidelines to ensure physiological relevance and reproducibility.

Aliquots were collected after each phase for phenolic content determination. Antioxidant activity was assessed only after the intestinal phase. All phases were performed in triplicate, on two independent days. For result calculations, the standard candy (without extract addition) was used as a blank, and its value was subtracted from the other groups for correction.

#### Bioaccessibility of phenolic compounds

The bioaccessibility of phenolic compounds was calculated as the amount of phenolic compounds in the aliquot collected at the end of the intestinal phase, referred to as the soluble fraction, relative to the amount of phenolic compounds found in the undigested sample, multiplied by 100, according to the following formula:^23^

$$\text{Bioaccessibility}\;\left(\%\right)=\frac{\text{Soluble fraction}}{\text{Undigested sample}}\;\times \;100$$

### Statistical analyses

All determinations were made at least in triplicate, and the final values were expressed as mean ± standard deviation (SD). The analysis results were subjected to analysis of variance (ANOVA) and the study of differences between means, detected by the Tukey test. Statistical analyses were performed using GraphPad Prism, version 6.0 (GraphPad Software Inc., San Diego, CA, USA). Results were considered statistically significant at *P* < 0.05.

## RESULTS AND DISCUSSION

### Characterization of peanut skin extract

The quantification of phenolic compounds in the peanut skin extract was performed using the Folin–Ciocâlteu method, resulting in a concentration of 0.473 mg GAE mg^−1^ dry extract. Furthermore, the antioxidant capacity determined by the ORAC method was 3.087 mmol TE mg^−1^. The identification of phenolic compounds in the peanut skin extract was previously performed using ultra‐performance liquid chromatographic–electrospray ionization–quadrupole time‐of‐flight–tandem mass spectrometric analysis,^12^ which revealed protocatechuic acid, coumaric acid, catechin and kaempferol as the major constituents.”

### Physicochemical characterization of pectin and gelatin gummy candies

Physicochemical analyses were conducted on both pectin‐ and gelatin‐based gummy candies, with detailed results presented in Table 2.

**Table 2:** Water activity, moisture and °Brix values of pectin and gelatin gummy candies in different compositions

Analyses	Pectin			Gelatin			
	P0	P1	P2	G0	G1	G2
Water activity	0.57a ± 0.0137	0.54b ± 0.0062	0.53b ± 0.0011	0.61a ± 0.0083	0.58b ± 0.0041	0.58b ± 0.0119
Moisture (%)	13.0b ± 0.9	14.0ab ± 0.3	15.2a ± 0.2	21.0a ± 0.5	19.0b ± 0.6	18.0b ± 0.3
°Brix	89a ± 0.2	89a ± 0.2	88b ± 0.1	80a ± 1.1	81a ± 0.2	77b ± 0.2
Hardness (g)	863.49a ± 24.09	754.58b ± 20.89	705.92b ± 7.09	521.42a ± 7.13	574.75b ± 9.89	656.32c ± 8.35
Viscosity (g)	−356.43a ± 36.37	−412.64a ± 12.98	−412.41a ± 11.52	−44.73a ± 8.43	−70.76b ± 3.55	−77.09b ± 5.62
L*	56.02a ± 4.36	33.70b ± 1.59	29.15c ± 1.02	51.41a ± 2.32	31.52b ± 3.21	33.33b ± 1.69
a*	−0.34a ± 0.14	9.85b ± 2.19	10.47b ± 5.00	−0.16a ± 0.23	7.73b ± 0.84	9.30c ± 1.69
b*	17.29a ± 1.62	7.14b ± 0.96	4.56c ± 1.80	18.95a ± 2.27	6.32b ± 0.92	7.39b ± 1.63

Mean ± standard deviation. Different letters within the same row indicate significant differences (*P* < 0.05). L* represents luminosity/brightness; a* denotes the red/green coordinate; b* denotes the yellow/blue coordinate. P0, pectin candy without extract; P1, pectin candy with 0.1 g kg^−1^ extract; P2, pectin candy with 0.2 g kg^−1^ extract; G0, gelatin candy without extract; G1, gelatin candy with 0.1 g kg^−1^ extract; G2, gelatin candy with 0.2 g kg^−1^ extract.

**Figure 2:**
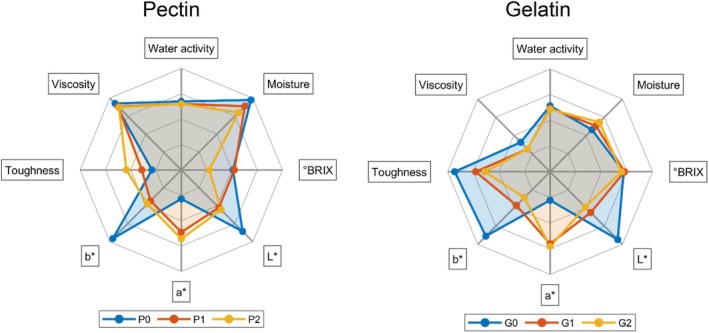
Comparison of the physicochemical results. Pectin candies: control (P0), 0.1 g kg^−1^ extract (P1) and 0.2 g kg^−2^ extract (P2); gelatin candies: control (G0), 0.1 g kg^−1^ extract (G1) and 0.2 g kg^−2^ extract (G2). Values are presented as means.

Figure 2 visually represents these data, highlighting the distinct physicochemical profiles of the two candy matrices. The radar charts illustrate that each matrix responded differently to the addition of the extract. Notably, pectin‐based candies demonstrated greater stability upon extract incorporation, as their physicochemical parameters exhibited less variation compared to the control. This enhanced stability may be attributed to the ability of pectin to form a more robust gel matrix, which likely contributes to better retention of bioactive compounds while preserving the structural integrity of the product.^24^

Regarding moisture, P2 candies had higher values (15.2%) than the control P0 (13.0%), while P1 showed no significant difference. In gelatin candies, G0 had moisture around 20%, which decreased with the addition of extract in G1 and G2. These results suggest that the extract influenced water retention differently in each matrix, possibly affecting other physicochemical properties such as °Brix and texture, as discussed below.

Water activity, or relative vapor pressure, is an important parameter often used to describe microbial stability, texture and water migration during storage.^25^ Our results regarding water activity showed that both pectin and gelatin‐based confections are in the range of 0.5–0.6, indicating water activity within the ideal and safe range in the literature (0.5–0.7).^26^ Besides, there was a statistical difference (*P* < 0.05) between the standard candy and candies with the addition of extract; however, no significant difference was found between the products with extract.

Regarding °Brix, a parameter used to determine the concentration of soluble solids, candies with higher extract content (P2 and G2) showed lower total soluble solids than the control candies (P0 and G0) (*P* < 0.05). In comparison, candies with lower extract content (P1 and G1) presented values similar to those of the controls. During the candy preparation process, those enriched with extract required a longer time in starch molds to reach the minimum necessary °Brix (75 °Brix). In addition, the candies enriched with extract did not dry uniformly. In the case of pectin candies, a moister layer formed on the outside, while the inside remained hard. Conversely, in gelatin candies, the opposite occurred, with a drier layer on the surface and a moister texture inside. This difference in drying may have influenced the moisture of the candies mentioned before. Despite that, the candies developed in this study fall within what is considered ideal by gum candy manufacturers,^27^ as both pectin‐ and gelatin‐based candies must contain at least 75 °Brix.

The extract's effect on hardness (force to compress) and viscosity (negative force during probe retraction), which relate to texture, is shown in Table 2 and Fig. 2. Peanut extract softened pectin candies (P1 and P2) compared to the control (P0) (*P* < 0.05) but did not change viscosity. Gelatin candies showed significant differences in both parameters, with G2 being harder than G1. These results may be due to low drying homogeneity (28) and interactions between the extract and gelatin protein affecting matrix rigidity and texture.

Thus, our results show that the peanut skin extract was able to influence the texture properties of the candies when compared to the standard candy, affecting the interaction between pectin and gelatin chains, decreasing the hardness value in pectin candies and increasing it in gelatin candies. In a previous study, the higher addition of peppermint extract was responsible for the viscosity alteration in gelatin candies.^28^ The difference in water activity and moisture directly influences the texture properties and stability of gels through polymeric bonds, as well as the addition of extract.^29^

When performing the CIELAB colorimetry test, both pectin and gelatin candies enriched with extract showed a significant difference in the three analyzed parameters (L*, a* and b*) when compared to the standard candy. The luminosity parameter values (L*), responsible for luminance ranging from white to black, decreased significantly for each formulation (P0 > P1 > P2 and G0 > G1 > G2), indicating that the higher the extract content, the lower the luminosity.

**Figure 3:**
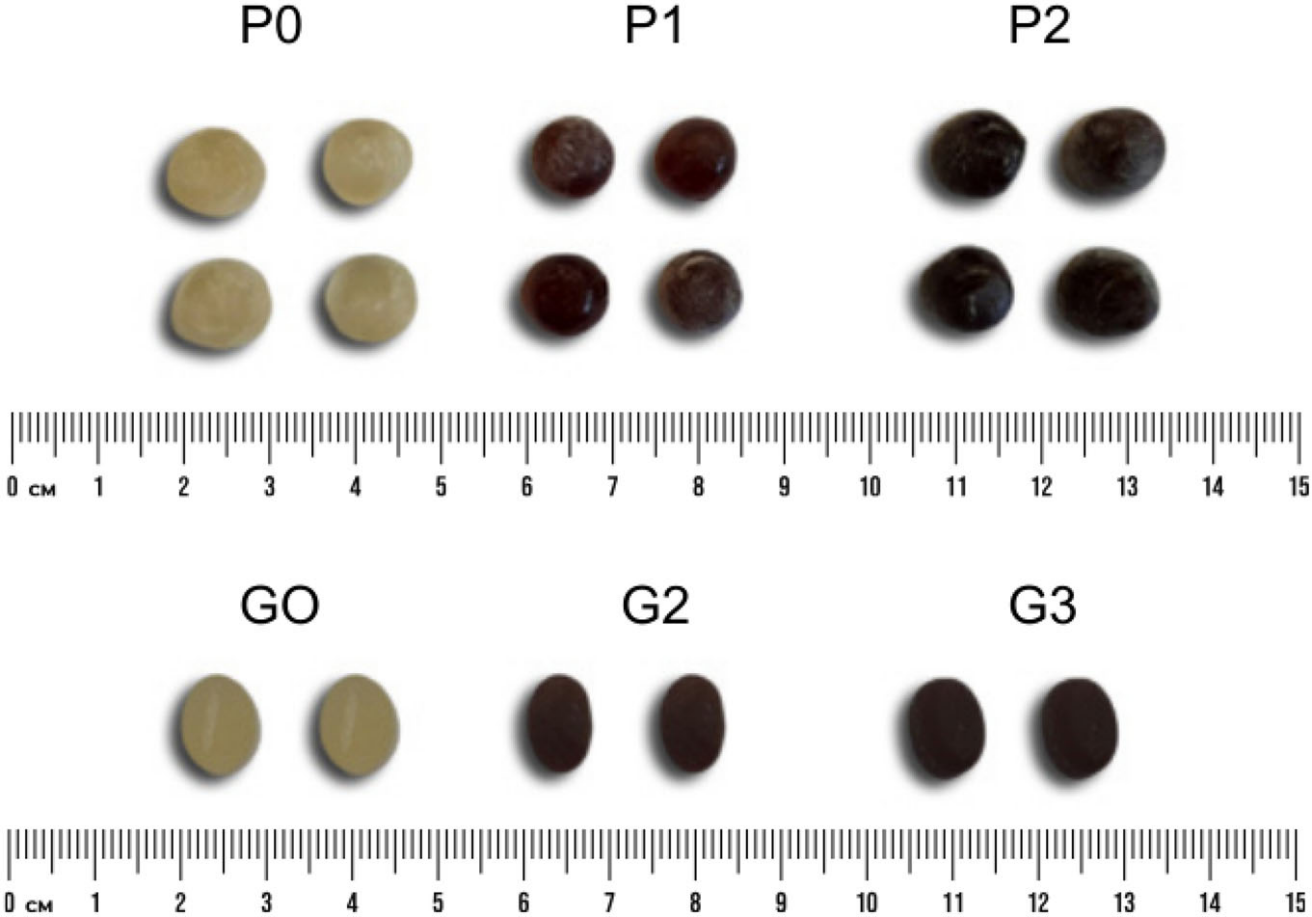
Pectin candies: control (P0), 0.1 g kg^−1^ extract (P1) and 0.2 g kg^−2^ extract (P2); gelatin candies: control (G0), 0.1 g kg^−1^ extract (G1) and 0.2 g kg^−2^ extract (G2).

In the chromatic analysis, the a* index (green–red axis) showed positive values, indicating a more reddish hue in candies enriched with extract. The b* index (blue–yellow axis) was higher in the standard candy, reflecting a more yellowish color. These results were stronger than expected, as the reddish‐brown peanut skin extract notably altered the candies' appearance at different concentrations (Fig. 3). This highlights the extract's potential as a natural colorant, adding value to its use in confections.

Health concerns about artificial colorants are driving the confectionery industry toward natural alternatives. These not only address health issues but also satisfy consumer demand for cleaner products. Similarly, gummy candies enriched with spirulina and açaí,^30^ and candies made with beetroot extract via cyclodextrin‐assisted ultrasound,^31^ showed colors comparable to those in this study. Additionally, color stability is closely linked to the stability of enriching compounds,^32^ underscoring its importance for product quality and consumer acceptance, since color strongly influences choice.

### Phenolic content of the candies and their bioaccessibility

**Figure 4:**
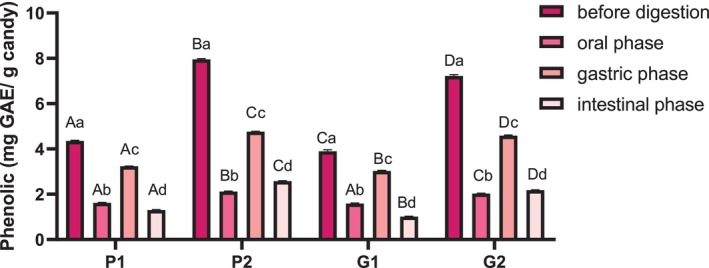
Total phenolic compounds in pectin and gelatin candies containing peanut skin extract, before digestion and after each *in vitro* phase: oral, gastric and intestinal. Results are expressed as mean ± standard error (*n* = 3). Different letters indicate significant differences (*P* < 0.05): upper‐case letters compare samples within the same digestive phase, lower‐case letters compare values within the same sample across digestive phases. Pectin candies: control (P0), 0.1 g kg^−1^ extract (P1), 0.2 g kg^−1^ extract (P2); gelatin candies: control (G0), 0.1 g kg^−1^ extract (G1), 0.2 g kg^−1^ extract (G2). GAE, gallic acid equivalents.

When evaluating the phenolic compounds in candies made with pectin and gelatin, using different concentrations of extract, lower values than expected were observed (between 8% and 24% less phenolics than what was expected; Fig. 4). Among the possible explanations for this reduction, our central hypothesis is the heating of the extracts during candy production, since syrups reach temperatures of up to 100 °C during cooking, which can cause degradation, oxidation or polymerization of phenolic compounds. Additionally, interactions with other candy ingredients, such as proteins (gelatin) and carbohydrates (sugar and glucose syrup), may lead to complex formation, further reducing total phenolic content.^33^, ^34^ Oxidation during storage may also contribute to this decrease.^35^

Besides, Fig. 4 shows that gelatin candies retained fewer total phenolics compared to pectin ones. Pectin may enhance phenolic stability by forming hydrogen bonds between its carboxyl groups and the hydroxyl groups of phenolic compounds, influencing their solubility and release.^36^ In contrast, gelatin can interact with phenolic compounds through electrostatic interactions, hydrogen bonding and hydrophobic forces, which may lead to complex formation affecting the solubility and bioavailability of phenolics.^37^, ^38^ These protein–polyphenol interactions can induce conformational changes in the protein structure, potentially altering antioxidant activity by masking or exposing phenolic functional groups.^39^ Understanding these interactions is crucial for optimizing the delivery and efficacy of bioactive compounds in food matrices.

After the *in vitro* digestion process of the gummy candies, the total phenolic content was quantified, followed by the determination of bioaccessibility in each phase (oral, gastric and intestinal). During the oral phase, pectin candies P1 (0.1 g kg^−1^ extract) released 37.2–40.8% of total phenolics compared to P0, while P2 (0.2 g kg^−1^ extract) released 26.6–28.1%. Similarly, gelatin candies G1 and G2 showed comparable trends. By the end of the gastric phase, phenolic release increased, reaching approximately 70% of total content, indicating optimal polyphenol release. However, in the intestinal phase, a significant reduction occurred, resulting in bioaccessibility levels of 26.0–32.5% for all candies.

These findings highlight that some phenolic compounds present in the candies reached their optimal release during the gastric phase but may have undergone degradation during the intestinal phase, reducing their availability for absorption. This reduction can be attributed to several factors, including the alkaline pH of the duodenum, enzymatic activity (such as bile salts and pancreatic enzymes) and micelle formation, which can affect phenolic stability and solubility. These processes may lead to diminished retention and bioaccessibility of phenolic compounds during digestion.^40^, ^41^, ^42^

To mitigate this reduction, encapsulation techniques and other advanced delivery systems have been proposed in the literature to protect phenolic compounds from degradation and improve their bioavailability.^43^ Despite the decrease observed, the bioaccessibility values found align with previously reported data, supporting the role of the gastrointestinal tract in promoting the release of phenolics from solid food matrices.^44^, ^45^

In an *in vitro* study, 77% of polyphenols were released after the gastric phase and 44% by the end of the intestinal phase.^46^ Another study investigated different chocolates enriched with phenolic compounds after simulated digestion and also observed a reduction in polyphenol content after the intestinal phase, finding approximately 23% of bioaccessible phenolics – a value even lower than that found in the present study.^47^

Pectin and gelatin gummy candies are considered simple food matrices from which phenolic compounds can be easily released during the digestion process, thus providing a favorable antioxidant environment in the digestive tract.^48^ Overall, pectin gummy candies exhibited better performance in releasing phenolic compounds across all gastrointestinal phases, except for the oral phase in P1 (0.1 g kg^−1^ extract) candies.

It is worth noting that, despite the doubled extract concentration in P2 and G2 (0.2 g kg^−1^) candies, the values obtained for phenolic compounds were not twice as high as those in P1 and G1 (0.1 g kg^−1^) candies. As highlighted,^49^ the amount of bioaccessible dietary polyphenols can differ from that obtained through chemical extraction, meaning that the most bioaccessible polyphenols are not necessarily those present in the highest concentrations in the food. Higher concentrations may result in more complex interactions within the food matrix, such as protein–polyphenol or carbohydrate–polyphenol binding, that can hinder the release and absorption of phenolic compounds. Studies have also shown significantly lower phenolic recovery in protein‐rich matrices, supporting the importance of matrix effects on bioaccessibility.^50^

Although it is essential to study the bioavailability of phenolic compounds to understand their health effects, a reduction during digestion does not imply a total loss of benefits. Digestion products may still be bioactive and serve as substrates for the intestinal microbiota.^51^, ^52^ In this study, despite the decrease in phenolic content, antioxidant activity persisted, suggesting potential biological effects post‐digestion.

### Antioxidant capacity of the candies before and after *in vitro* gastrointestinal digestion

**Figure 5:**
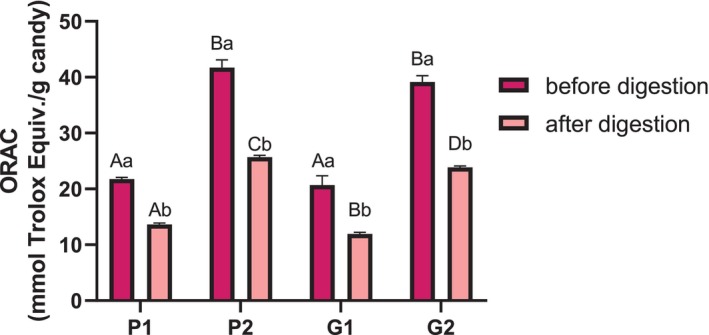
*In vitro* antioxidant activity of pectin and gelatin gummies enriched with peanut skin extract, measured before and after *in vitro* digestion. Results are presented as mean ± standard error (*n* = 3). Different letters indicate statistically significant differences (*P* < 0.05). Upper‐case letters indicate differences between samples within the same digestive phase, while lower‐case letters indicate differences within the same sample across digestive phases. Pectin candies: control (P0), 0.1 g kg^−1^ extract (P1), 0.2 g kg^−1^ extract (P2); gelatin candies: control (G0), 0.1 g kg^−1^ extract (G1), 0.2 g kg^−1^ extract (G2). ORAC, oxygen radical absorbance capacity; TE, Trolox equivalents.

Figure 5 shows that the candy matrix (pectin or gelatin) did not affect the *in vitro* antioxidant activity, as candies with peanut extract had similar ORAC values, regardless of the matrix. After digestion, ORAC values dropped significantly – about 38% for pectin candies and 41% for gelatin ones – reflecting the expected reduction in phenolic compounds.

Besides, when comparing the antioxidant capacity between digested pectin and gelatin candies (Fig. 5), gelatin exhibited lower antioxidant activity than pectin candies. This difference may be due to the difference in the food matrix of the candies. Although gelatin is not capable of forming strong complexes with phenolics, weak interactions between gelatin proteins and phenolic compounds may still occur. These interactions can result in some changes in the properties of phenolics, such as a decrease in their antioxidant capacity.^53^, ^54^

After subjecting gelatin candies enriched with propolis extract to gastrointestinal digestion, a study identified 6.59 mmol TE kg^−1^ by ORAC analysis, corresponding to approximately 41.80% compared to undigested candies containing 0.1 g kg^−1^ propolis extract.^55^ This value is lower than that obtained in the present study, which found a rate of 57.83% relative to undigested gelatin candies at the same extract concentration.

The antioxidant capacity after *in vitro* digestion depends on the food matrix due to interactions between bioactive compounds and other food components.^56^ In a study with various types of candies enriched with blackcurrant, antioxidant capacity values varied in the intestinal phase, with higher values found in the non‐bioaccessible portion of the candies.^48^

It is essential to consider that some bioactive compounds may exert beneficial effects locally in the stomach and intestines, organs constantly exposed to reactive oxygen species (ROS). Even without systemic absorption, these compounds can contribute to maintaining redox balance and may help prevent gastrointestinal diseases linked to ROS generated during digestion.^57^

In this context, the antioxidant capacity of peanut extract‐enriched gummy candies highlights their potential as functional confectionery products. According to the United States Department of Agriculture (USDA), fresh blueberries contain approximately 24 mmol TE kg^−1^. A 20 g serving of pectin‐based gummies with 0.1 and 0.2 g kg^−1^ extract showed antioxidant activity equivalent to approximately 18 g and 35 g of fresh blueberries, respectively. Gelatin‐based gummies with the same extract levels showed similar values. These results confirm the significant contribution of peanut extract to the antioxidant properties of the gummies.

## CONCLUSION

The incorporation of peanut skin extract, a rich source of phenolic compounds with antioxidant activity, into gummy candies showed positive results in both pectin and gelatin matrices. Although digestion reduced the phenolic content, pectin‐based candies retained a higher proportion and exhibited greater antioxidant capacity after digestion, which may be related to the formation of stable complexes. Encapsulation could be further explored as a strategy to protect these compounds during gastrointestinal transit. Future studies should evaluate protection strategies such as micro‐ or nano‐encapsulation (e.g., spray‐drying, complex coacervation, liposomes or cyclodextrin inclusion) and process optimizations (reduced thermal exposure and adjusted formulation) to improve phenolic stability and bioaccessibility.

The observed antioxidant activity after digestion suggests that these candies can deliver bioactive compounds with the potential to contribute to oxidative stress reduction, within the limits of the studied model. Overall, the findings indicate that gummy candies containing peanut shell extract may serve as a functional confectionery option, combining a source of bioactive compounds with a familiar and enjoyable product format. In addition to their potential antioxidant benefits, this approach also supports the valorization of a low‐cost, sustainable by‐product.

## Data Availability

Research data are not shared.
